# Humoral and cellular immunogenicity to a second dose of COVID-19 vaccine BNT162b2 in people receiving methotrexate or targeted immunosuppression: a longitudinal cohort study

**DOI:** 10.1016/S2665-9913(21)00333-7

**Published:** 2021-11-09

**Authors:** Satveer K Mahil, Katie Bechman, Antony Raharja, Clara Domingo-Vila, David Baudry, Matthew A Brown, Andrew P Cope, Tejus Dasandi, Carl Graham, Hataf Khan, Thomas Lechmere, Michael H Malim, Freya Meynell, Emily Pollock, Kamila Sychowska, Jonathan N Barker, Sam Norton, James B Galloway, Katie J Doores, Timothy Tree, Catherine H Smith

**Affiliations:** a St John's Institute of Dermatology, Guy's and St Thomas' NHS Foundation Trust, London, UK; b St John's Institute of Dermatology, Faculty of Life Sciences and Medicine, King's College London, London, UK; c Department of Immunobiology, Faculty of Life Sciences and Medicine, King's College London, London, UK; d Centre for Rheumatic Diseases, King's College London, London, UK; e Department of Infectious Diseases, School of Immunology and Microbial Sciences, King's College London, London, UK; f Psychology Department, Institute for Psychiatry Psychology and Neuroscience, King's College London, London, UK

## Abstract

**Background:**

COVID-19 vaccines have robust immunogenicity in the general population. However, data for individuals with immune-mediated inflammatory diseases who are taking immunosuppressants remains scarce. Our previously published cohort study showed that methotrexate, but not targeted biologics, impaired functional humoral immunity to a single dose of COVID-19 vaccine BNT162b2 (Pfizer-BioNTech), whereas cellular responses were similar. Here, we aimed to assess immune responses following the second dose.

**Methods:**

In this longitudinal cohort study, we recruited individuals with psoriasis who were receiving methotrexate or targeted biological monotherapy (ie, tumour necrosis factor [TNF] inhibitors, interleukin [IL]-17 inhibitors, or IL-23 inhibitors) from a specialist psoriasis centre serving London and South-East England. The healthy control cohort were volunteers without psoriasis, not receiving immunosuppression. Immunogenicity was evaluated immediately before, on day 28 after the first BNT162b2 vaccination and on day 14 after the second dose (administered according to an extended interval regimen). Here, we report immune responses following the second dose. The primary outcomes were humoral immunity to the SARS-CoV-2 spike glycoprotein, defined as titres of total spike-specific IgG and of neutralising antibody to wild-type, alpha (B.1.1.7), and delta (B.1.617.2) SARS-CoV-2 variants, and cellular immunity defined as spike-specific T-cell responses (including numbers of cells producing interferon-γ, IL-2, IL-21).

**Findings:**

Between Jan 14 and April 4, 2021, 121 individuals were recruited, and data were available for 82 participants after the second vaccination. The study population included patients with psoriasis receiving methotrexate (n=14), TNF inhibitors (n=19), IL-17 inhibitors (n=14), IL-23 inhibitors (n=20), and 15 healthy controls, who had received both vaccine doses. The median age of the study population was 44 years (IQR 33–52), with 43 (52%) males and 71 (87%) participants of White ethnicity. All participants had detectable spike-specific antibodies following the second dose, and all groups (methotrexate, targeted biologics, and healthy controls) demonstrated similar neutralising antibody titres against wild-type, alpha, and delta variants. By contrast, a lower proportion of participants on methotrexate (eight [62%] of 13, 95% CI 32–86) and targeted biologics (37 [74%] of 50, 60–85; p=0·38) had detectable T-cell responses following the second vaccine dose, compared with controls (14 [100%] of 14, 77–100; p=0·022). There was no difference in the magnitude of T-cell responses between patients receiving methotrexate (median cytokine-secreting cells per 10^6^ cells 160 [IQR 10–625]), targeted biologics (169 [25–503], p=0·56), and controls (185 [133–328], p=0·41).

**Interpretation:**

Functional humoral immunity (ie, neutralising antibody responses) at 14 days following a second dose of BNT162b2 was not impaired by methotrexate or targeted biologics. A proportion of patients on immunosuppression did not have detectable T-cell responses following the second dose. The longevity of vaccine-elicited antibody responses is unknown in this population.

**Funding:**

NIHR Biomedical Research Centre at Guy's and St Thomas' NHS Foundation Trust and King's College London; The Psoriasis Association.

## Introduction

Individuals with immune-mediated inflammatory diseases were shown to be at increased risk of COVID-19-related death compared with the general population.^1^ Based on previous research in other infections and registry data collected during the COVI-19 pandemic,^2^, ^3^, ^4^ there has been concern over the detrimental effect of systemic immunosuppressants (the mainstay of treatment of moderate to severe immune-mediated inflammatory diseases) on COVID-19 outcomes. Vaccination is therefore a vital risk mitigating strategy in this population. General population-based studies indicate that two doses of a COVID-19 vaccine provides protection against SARS-CoV-2 infections and related adverse outcomes.^5^, ^6^ However, immunogenicity and clinical effectiveness in patients with immune-mediated inflammatory diseases who are receiving immunosuppressants remains uncertain. These patients were excluded from COVID-19 vaccine trials and most previous studies have focused on seroconversion alone,^7^, ^8^, ^9^, ^10^ which might not adequately reflect vaccine immunogenicity or correlate with effectiveness.

**Research in context**

**Evidence before this study**

Two COVID-19 vaccine doses, administered according to an extended interval regimen, are highly efficacious at protecting against SARS-CoV-2 infections and adverse COVID-19 outcomes in the general population. However, data for individuals with immune-mediated inflammatory diseases who are taking immunosuppressants remain scarce. Understanding vaccine immunogenicity in this vulnerable population is of major importance to public and individual health. The interim analysis of our cohort study at day 28 (after one dose) showed that methotrexate but not targeted biologics impaired functional humoral immunity (ie, neutralising antibody responses) to the first dose of the COVID-19 vaccine BNT162b2 (Pfizer-BioNTech), whereas T-cell responses were similar. The OCTAVE study, involving a heterogeneous cohort of patients with immune-mediated inflammatory diseases, indicated lower serological and similar T-cell responses to two vaccine doses, compared with healthy controls. No medication-specific effects or functional humoral responses were investigated in the OCTAVE study. No studies have concurrently assessed immunosuppressant-specific effects on serological, neutralising antibody (including against the delta variant) and cellular responses to a second vaccine dose, administered at an extended interval, in patients with immune-mediated inflammatory diseases.

**Added value of the study**

We evaluated the effect of methotrexate and biologics targeting tumour necrosis factor, interleukin (IL)-17, and IL-23 on humoral (serological and functional) and cellular immune responses to the second dose of the COVID-19 vaccine BNT162b2 in individuals with psoriasis. All patients receiving immunosuppressants and all healthy controls seroconverted (ie, had detectable spike-specific IgG) at 14 days following the second dose. Neutralising antibody titres against wild-type SARS-CoV-2, alpha (B.1.1.7), and delta (B.1.617.2) variants were similar in patients receiving methotrexate, targeted biologics, and healthy controls. Whereas a proportion of healthy controls and patients had undetectable spike-specific T-cell responses (interferon-γ, IL-2, or IL-21) after the first dose, all controls had detectable T-cell responses at 14 days after the second dose. By contrast, a proportion of patients receiving immunosuppressants had undetectable spike-specific T-cell responses following the second dose (similar to after the first dose). The magnitude of response was similar in patients receiving methotrexate, targeted biologics, and controls.

**Implications of all the available evidence**

These findings indicate that functional humoral immunity at 14 days after a second dose of the BNT162b2 COVID-19 vaccine was not impaired by methotrexate or targeted biologics. A proportion of patients on immunosuppression did not have detectable T-cell responses after the second dose. The durability of antibody responses in the context of undetectable cellular responses is uncertain, underscoring the importance of ongoing risk mitigation efforts and research into further vaccine doses in this population.

Several countries, including the UK, adopted an extended interval vaccination regimen (up to 12 weeks between doses) to maximise population coverage with a single (prime) vaccine. Compared with the standard 3 week interval, the extended interval regimen has been shown to increase peak antibody responses and improve vaccine efficacy in the general population.^11^, ^12^ The interim findings at day 28 (after one dose) of our longitudinal cohort study of patients with psoriasis showed that methotrexate, but not targeted biological monotherapy, impaired functional humoral immunity in response to a single dose of BNT162b2 (Pfizer-BioNTech), whereas cellular responses were similar, between patients with psoriasis and healthy controls.^13^ Emerging larger-scale research in heterogeneous cohorts of patients with immune-mediated inflammatory diseases indicated lower serological and similar cellular responses to two vaccine doses, compared with healthy controls.^14^ However, there is a paucity of data on medication-specific effects on the immunogenicity of a second vaccine dose,^15^ including against novel, highly transmissible, SARS-CoV-2 variants of concern.

We sought to assess the effect of methotrexate and targeted biological monotherapy on early humoral and cellular immune responses after the second dose of BNT162b2 in patients with psoriasis who were vaccinated at an extended dosing interval. We also aimed to gain insight into immunity against the predominant delta variant (B.1.617.2), which has emerged since our interim analysis and presents a challenge to vaccination strategies.^16^, ^17^

## Methods

### Study design and participants

We conducted a prospective, longitudinal cohort study in individuals with a dermatologist-confirmed diagnosis of psoriasis who were receiving methotrexate or targeted biological monotherapy (tumour necrosis factor [TNF] inhibitors, interleukin [IL]-17 inhibitors, or IL-23 inhibitors). The healthy control cohort were volunteers without psoriasis and not receiving systemic immunosuppression. We recruited all individuals from a specialist psoriasis centre (Severe Psoriasis Service, St John's Institute of Dermatology, Guy's and St Thomas' NHS Foundation Trust, London, UK) serving London and South-East England. We excluded individuals from the main analysis if they had previous SARS-CoV-2 infection (serologically confirmed) or had received the ChAdOx1 nCoV-19 vaccine, as described previously.^13^ This study was approved by the London Bridge Research Ethics Committee (REC reference 06/Q0704/18). All participants provided written informed consent before enrolment.

### Procedures

Clinical and safety data and blood samples were collected at three study visits: baseline (same day of and immediately before vaccination), 28 days after the first dose of BNT162b2 (±2 days), and 14 days (±2 days) after the second dose. The second dose was administered according to the UK Government extended interval vaccine policy of up to 12 weeks. Immune responses following the first dose were previously described.^13^ Here, we report findings following the second vaccine dose.

Plasma and peripheral blood mononuclear cells were isolated from blood samples and processed as previously described.^13^ Humoral immunity was based on seroconversion, assessed using ELISAs for IgG specific for the SARS-CoV-2 spike glycoprotein and the functional capacity of participants' plasma to neutralise the HIV-1 viral particles pseudotyped with spike of prototypic (wild-type) strain of SARS-CoV-2, alpha (B.1.1.7) variant, or delta (B.1.617.2) variant.^18^ Seroconversion was defined as IgG four-fold above the background at the first dilution point (1:25). A threshold half maximal effective concentration (EC_50_) was used for anti-SARS-CoV-2 IgG titres, at which serological responses were classified as positive. Neutralisation activity is presented as 50% inhibitory dilution (ID_50_). Vaccine-induced cellular immunity was determined via spike-specific T-cell production of interferon-γ and IL-2 (type 1 helper T cell [Th1] cytokines), and IL-21 (T follicular helper cell [Tfh] cytokine). These responses were quantified following stimulation with two peptide pools spanning the entire length of the SARS-CoV-2 spike glycoprotein. Direct FluoroSpot assays were used to detect interferon-γ and IL-2, and direct ELISpot assays were used to detect IL-21. Receiver operator characteristic curve analysis was used to establish threshold values to determine a positive response for the total T-cell response (combined interferon-γ, IL-2, and IL-21) and for each individual cytokine. A threshold value of 30 cytokine-secreting cells per million peripheral blood mononuclear cells was established, at which level the total T-cell response was classified as positive.

### Outcomes

The primary outcomes were humoral immunity to the SARS-CoV-2 spike glycoprotein 14 days after the second dose of BNT162b2, defined as neutralising antibody responses to wild-type, alpha (B.1.1.7), and delta (B.1.617.2) SARS-CoV-2 variants, and spike-specific T-cell responses (ie, production of interferon-γ, IL-2, IL-21, or a combination thereof). The key exposure measure was immunosuppressive treatment type. We tested two predetermined hypotheses: first, that after the second dose of BNT162b2, patients taking methotrexate would have lower humoral immunity, cellular immunity, or both compared with healthy controls; and second, that patients taking methotrexate would have lower humoral immunity, cellular immunity, or both compared with patients taking targeted biological therapy (TNF inhibitors, IL-17 inhibitors, or IL-23 inhibitors).

Adverse events were classified as local or systemic, and graded as mild (no or minimal interference with participants' activity and no or minimal medical intervention required), moderate (limitation in activity and medical intervention required), severe (marked limitation in activity and medical intervention required).

### Statistical analysis

Demographic and clinical characteristics and humoral and cellular immunogenicity were summarised using descriptive statistics. Statistical imbalance across the treatment groups was calculated with the Kruskal-Wallis or χ^2^ test. Further comparisons were made using a linear regression model with robust estimates of variance to derive 95% CIs and p values. The regression models incorporated log transformed immunogenicity measures as the dependent variable, and methotrexate versus healthy controls or methotrexate versus biologics as a binary independent variable. We adjusted for age and sex. Model diagnostics were evaluated to check for heteroskedasticity and linearity assumptions. A 5% α level was used for significance testing. The study was exploratory in nature, and therefore no correction for multiple hypothesis testing was made. Data were analysed in Stata (version 16).

### Role of the funding source

The funders of the study had no role in study design, data collection, data analysis, data interpretation, or writing of the report.

## Results

**Figure 1:**
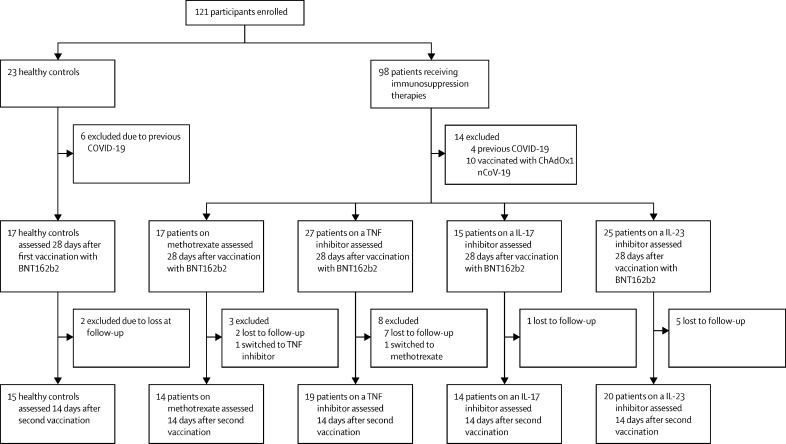
Study overview IL=interleukin. TNF=tumour necrosis factor.

Between Jan 14 and April 4, 2021, 121 individuals were enrolled, of whom 39 (32%) were excluded from the final analysis on the basis of immunisation with the ChAdOx1 nCoV-19 vaccine (n=10), previous history of COVID-19 (n=10), switching immunosuppression therapy between the first and second dose (n=2; one individual switched from methotrexate monotherapy to adalimumab monotherapy, and the second individual switched from certolizumab monotherapy to cotherapy with certolizumab and methotrexate), or loss to follow up after the first vaccine dose (n=17; figure 1). The final analysis (n=82) included 67 individuals with psoriasis receiving therapeutic immunosuppression, including methotrexate (n=14), TNF inhibitors (n=19), IL-17 inhibitors (n=14), IL-23 inhibitors (n=20), and 15 healthy volunteers (the control group).

The median age of the total study population was 44 years (IQR 33–52), with 43 (52%) males and 71 (87%) participants of White ethnicity (table 1, appendix p 1). Among the 67 patients, the median duration of psoriasis was 21 years (IQR 15–33); all had well controlled psoriasis, as indicated by their baseline disease severity. No participants receiving immunosuppression monotherapy reported a pause or stop in medication before or following BNT162b2 vaccination. Of those who were receiving methotrexate, the median dose of methotrexate was 15·0 mg (range 5·0–22·5 mg per week. No patients were receiving concomitant corticosteroid therapy. The time between the last dose of immunosuppression and vaccination was similar for vaccine doses 1 and 2 in all therapy groups (table 1). The median spacing between vaccine doses was 10·0 weeks (IQR 9·3–10·9); the control cohort had a slightly longer spacing between doses (11·0 weeks [10·3–11·4]) compared with those on immunosuppression (9·9 weeks [9·1–10·6]; p<0·0001).

**Table 1:** Baseline characteristics of study participants with data following both the first and second dose of COVID-19 vaccine BNT162b2

		**Control group (n=15)**	**Methotrexate group (n=14)**	**TNF inhibitor group (n=19)**	**IL-17 inhibitor group (n=14)**	**IL-23 inhibitor group (n=20)**	**Total (n=82)**	**p value**
Age, years		38·0 (27·0–47·0)	49·5 (43·0–57·0)	36·0 (27·0–52·0)	43·0 (38·0–48·0)	50·5 (35·5–59·0)	43·5 (33·0–52·0)	0·046
Sex								
	Female	7 (47%)	6 (43%)	11 (58%)	7 (50%)	8 (40%)	39 (48%)	..
	Male	8 (53%)	8 (57%)	8 (42%)	7 (50%)	12 (60%)	43 (52%)	0·84
BMI, kg/m^2^		24·0 (21·8–28·7)	27·9 (26·5–33·3)	30·3 (28·3–31·9)	27·3 (25·2–29·8)	28·7 (26·3–33·1)	28·5 (25·2–31·6)	0·11
Ethnicity								
	White	13 (87%)	11 (79%)	18 (95%)	12 (86%)	17 (85%)	71 (87%)	0·69
	Black	0 (0%)	1 (7%)	0 (0%)	0 (0%)	0 (0%)	1 (1%)	..
	South Asian	2 (13%)	2 (14%)	1 (5%)	2 (14%)	2 (10%)	9 (11%)	..
	Mixed	0 (0%)	0 (0%)	0 (0%)	0 (0%)	1 (5%)	1 (1%)	..
Disease severity measure, psoriasis area severity index		..	2·3 (0·8–3·9)	1·2 (0·8–3·0)	0·4 (0·0–1·5)	0·6 (0·0–2·8)	1·2 (0·0–2·8)	0·053
Concomitant psoriatic arthritis		..	2 (14%)	3 (16%)	7 (50%)	6 (30%)	18 (22%)	0·015
Proximity of methotrexate or biologic to vaccine dose 1, days		..	3·5 (1·0–5·0)	6·0 (5·0–10·0)	18·5 (11·0–21·0)	42·0 (23·5–48·0)	10·0 (5·0–23·0)	<0·0001
Proximity of methotrexate or biologic to vaccine dose 2, days		..	2·0 (1·0–5·0)	5·5 (1·0–7·0)	18·0 (9·0–22·0)	37·5 (24·0–60·0)	7·5 (3·0–23·0)	<0·0001
Time between vaccine doses, weeks		11·0 (10·3–11·4)	9·8 (9·4–10·0)	9·1 (9·0–10·4)	9·6 (9·1–10·0)	10·0 (9·7–10·6)	10·0 (9·3–10·9)	0·002

All values are given as n (%) or median (IQR), unless otherwise specified. Statistical imbalance of the baseline characteristics across the treatment groups presented by either Kruskal-Wallis or χ^2^. BMI=body-mass index. TNF=tumour necrosis factor. IL=interleukin.

No participants reported COVID-19 during the study. Mild adverse events after the second vaccine dose were reported by 42 (63%) of 67 patients receiving immunosuppression compared with 12 (80%) of 15 controls (appendix p 2, 5, 6). Adverse events of moderate severity were reported five times in patients with psoriasis after the second dose: headache (n=1), malaise (n=2), and fatigue (n=2). The commonest local adverse effect was pain at the injection site and the commonest systemic adverse event was fatigue (appendix pp 2, 5, 6). Worsening psoriasis was reported by eight (12%) of 67 (95% CI 5–22) patients after the second vaccination.

Data for humoral immunogenicity in response to the second dose were missing for three patients with psoriasis; one each receiving a TNF inhibitor, IL-17 inhibitor, and IL-23 inhibitor. Cellular data were missing for one healthy control and four patients; two on IL-23 inhibitors, one on methotrexate, and one on an IL-17 inhibitor.

**Figure 2:**
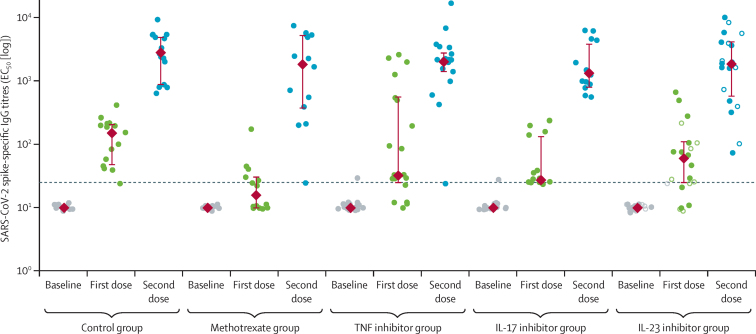
Serological immune responses to COVID-19 vaccine BNT162b2 Spike-specific IgG titres (EC_50_) in plasma samples at day 28 following the first dose and at day 14 following the second dose of COVID-19 vaccine BNT162b2 in healthy controls and patients with psoriasis receiving methotrexate or targeted biological monotherapy. The circles represent individual values. The red diamonds indicate the median and the range lines indicate IQR. In the IL23 inhibitor group, filled circles represent participants receiving IL-23p19 inhibitors and open circles represent participants receiving IL-23/IL-12p40 inhibition. The horizontal dashed line indicates the seroconversion threshold. EC_50_=half maximal effective concentration. IL=interleukin. TNF=tumour necrosis factor.

All 79 participants with humoral immunogenicity data (100% [95% CI 96–100]) seroconverted after the second BNT162b2 dose (table 2, figure 2). The second dose successfully boosted spike-specific IgG titres in all groups. The fold change in spike-specific IgG titre between doses 1 and 2 was highest in patients receiving methotrexate (median fold change 42 [IQR 23–159]) compared with healthy controls (20 [13–35]) and patients on targeted biologics (33 [15–73]).

**Table 2:** Efficacy of the first and second dose of COVID-19 vaccine BNT162b2 in study participants

		**Number of responders**	
		**Anti-SARS-CoV-2 IgG (serological) vaccine response**	**T cell vaccine response**
First vaccine dose		67/82	68/82
	Healthy controls	15/15 (100%)	10/15 (67%)
	Patients on immunosuppressants	52/67 (78%)	58/67 (87%)
	Patients on methotrexate	7/14 (50%)	13/14 (93%)
	Patients on TNF inhibitors	15/19 (79%)	15/19 (79%)
	Patients on IL-17 inhibitors	14/14 (100%)	13/14 (93%)
	Patients on IL-23 inhibitors	16/20 (80%)	17/20 (85%)
Second vaccine dose		79/79*	59/77†
	Healthy controls	15/15 (100%)	14/14 (100%)
	Patients on immunosuppressants	64/64 (100%)	45/63 (71%)
	Patients on methotrexate	14/14 (100%)	8/13 (62%)
	Patients on TNF inhibitors	18/18 (100%)	15/19 (79%)
	Patients on IL-17 inhibitors	13/13 (100%)	8/13 (62%)
	Patients on IL-23 inhibitors	19/19 (100%)	14/18 (78%)

Data are n (%). A threshold EC_50_ value of 25 was established for anti-SARS-CoV-2 IgG titres, at which serological responses were classified as positive. A threshold value of 30 cytokine secreting cells per million peripheral blood mononuclear cells was established for total T cell responses (IFNγ, IL-2, or IL-21), at which the T cell response was classified as positive.

* Serological data were missing for three patients after the second vaccine dose (one each receiving a TNF inhibitor, IL-17 inhibitor, and IL-23 inhibitor).

† T-cell data were missing for one participant in the control group and four patients after the second vaccine dose (two on IL-23 inhibitors, one on methotrexate, and one on an IL-17 inhibitor).

There was no statistically significant difference in spike-specific IgG titres following the second dose between patients receiving immunosuppressants (median EC_50_ 1816 [IQR 641–3645]) and healthy controls (2749 [867–4770], p=0·14; figure 2). Median titres were numerically lowest in patients receiving methotrexate (median EC_50_ 1751 [IQR 468–4976] compared with patients on targeted biologics (1816 [787–3534]; p=0·65) and healthy controls (2749 [867–4770]; p=0·20).

**Figure 3:**
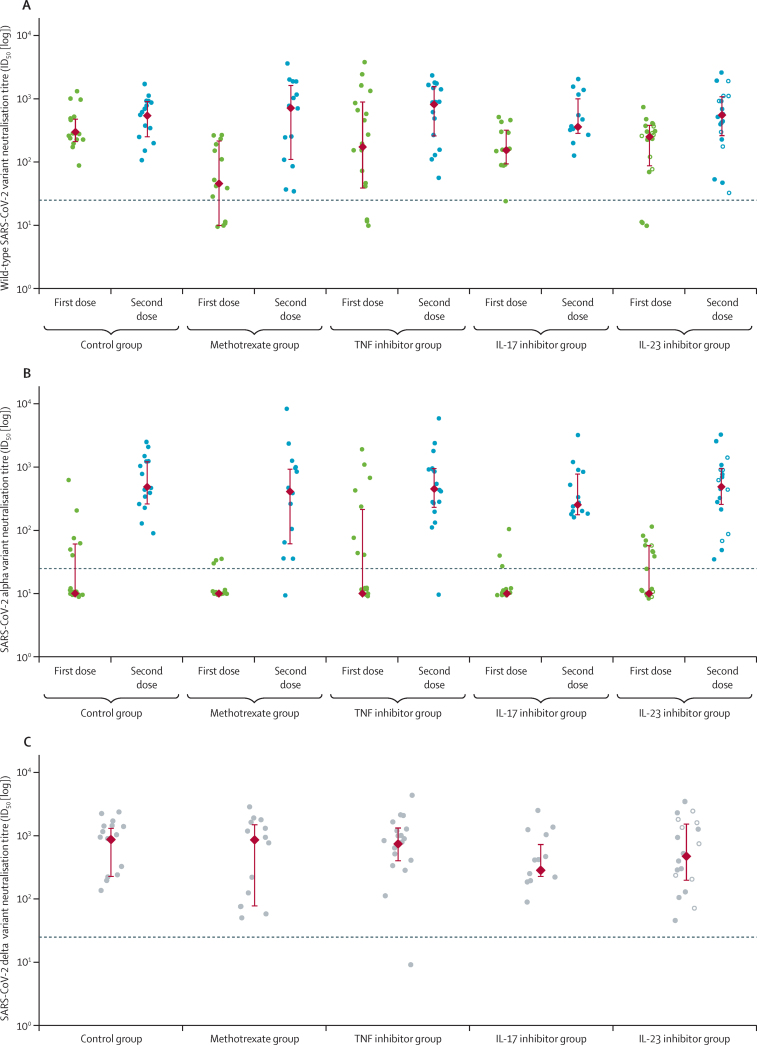
Functional humoral immunogenicity of COVID-19 vaccine BNT162b2 Neutralisation titres (ID_50_) against SARS-CoV-2 in plasma titres at day 28 following the first dose and at day 14 following the second dose of COVID-19 vaccine BNT162b2 in healthy controls and patients with psoriasis receiving methotrexate or targeted biological monotherapy. The red diamonds indicate the median and the range lines indicate IQR. In the IL23 inhibitor group, filled circles represent participants receiving IL-23p19 inhibitors and hollow circles represent participants receiving IL-23/IL-12p40 inhibition. The horizontal dashed line indicates neutralisation activity detection threshold. Neutralising antibody titres against wild-type SARS-CoV-2 (A). Neutralising antibody titres against alpha (B.1.1.7) variant (B); IQR bars are not visible for methotrexate and IL-17 inhibitor for the first dose data. Neutralising antibody titres against delta (B.1.617.2) variant (C), assessed at day 14 following the second dose only. The circles represent individual values. ID_50_=50% inhibitory dilution. IL=interleukin. TNF=tumour necrosis factor.

All study participants had detectable neutralising antibodies against wild-type SARS-CoV-2 following the second vaccine dose, and all groups showed increased neutralising antibody titres against wild-type SARS-CoV-2 and the alpha variant after the second dose compared to the first (figure 3A, B). The fold change in neutralising antibody titres against wild-type SARS-CoV-2 between the first and second dose was significantly higher in patients receiving methotrexate (median fold change 6·5 [IQR 4·2–13·6]) compared with healthy controls (1·4 [0·9–2·7], p=0·0006) and patients on targeted biologics (3·2 [1·5–5·3], p=0·023). Nearly all participants had a detectable neutralising antibody response against the alpha variant after the second vaccine dose (77 [97%] of 79), compared with 29 [36%] of 81 participants after the first dose. Data were missing for one individual after the first dose (from the IL-23 group) and three individuals after the second dose (from the IL-17, TNF, and IL-23 groups). Changes in neutralising activity against the delta variant between doses 1 and 2 could not be assessed due to the absence of data for the first dose.

All groups had similar neutralising antibody titres against wild-type SARS-CoV-2 following the second dose of BNT162b2 (figure 3A). Similarly, with respect to the alpha variant, patients receiving methotrexate (50% inhibitory dilution [ID_50_] 440 [IQR 101–935]) had similar neutralisation activity to those receiving targeted biological therapy (453 [233–955], p=0·97) and healthy controls (491 [264–1227], p=0·63; figure 3B). Neutralisation activity against the delta variant was also detected at similar levels across all groups; patients receiving methotrexate (ID_50_ 856 [77–1476]) compared with targeted biologics (469 [232–1309], p=0·76) and controls (863 [225–1297], p=0·49; figure 3C). There was no association between neutralising antibody titres and the time interval between the first and second dose of vaccine (appendix p 7).

There was no evidence of induction or boosting of total T cell responses (as evidenced by production of interferon-γ, IL-2, or IL-21) between the first and second vaccine doses in patients receiving methotrexate (median fold change of log cytokine production was 1·0 [IQR 0·4–1·3; appendix p 8). A similar lack of boosting was demonstrated in those taking targeted biologics (1·1 [0·7–1·3], p=0·54). By contrast, compared with those receiving methotrexate, healthy controls demonstrated a greater induction of T-cell responses (1·5 [IQR 1·0–4·0], p=0·033). Similar results were found on assessment of individual cytokines (appendix pp 3, 9).

Following the second vaccine dose, a significantly lower proportion of patients receiving immunosuppression (45 [71%] of 63 [95% CI 59–82]) had detectable T-cell responses compared with controls (14 [100%] of 14 [77–100], p=0·022; table 2). Thus, in contrast to our healthy population, nearly a third of patients on immunosuppression had no detectable T-cell response.

**Figure 4:**
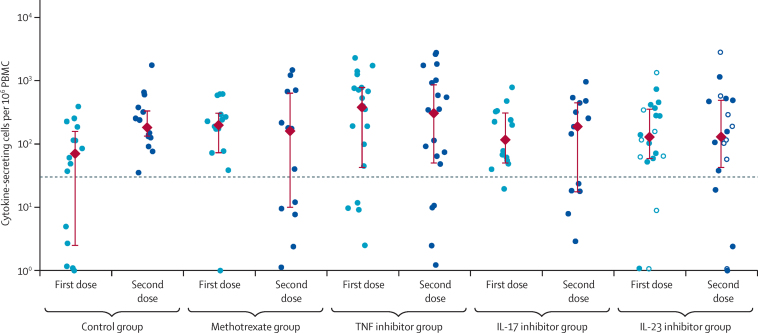
Cellular immunogenicity of COVID-19 vaccine BNT162b2 The red diamonds indicate the median and the range lines indicate IQR. Total T cell response, as determined by IFNγ, IL-2, or IL-21 responses to stimulation with peptides from total spike peptide pools, reported as number of cytokine secreting cells per 10^6^ PBMCs at day 28 following the first dose and at day 14 following the second dose of COVID-19 vaccine BNT162b2. The circles represent individual values. In the IL23 inhibitor group, filled circles represent participants receiving IL-23p19 inhibitors and hollow circles represent participants receiving IL-23p40/IL-12 inhibition. The horizontal dashed line indicates total T cell response threshold. IL=interleukin. PBMCs=peripheral blood mononuclear cells. TNF=tumour necrosis factor.

A slightly numerically lower proportion of patients receiving methotrexate (eight [62%] of 13 [95% CI 32–86]) had detectable T-cell responses compared with those receiving targeted biologics (37 [74%] of 50 [60–85], p=0·38) and controls (14 [100%] of 14 [77–100], p=0·022). There was no difference in the magnitude of T cell responses between patients receiving methotrexate (median log-cytokine-secreting cells per 10^6^ 160 [IQR 10–625]), targeted biologics (169 [25–503], p=0·56), and controls (185 [133–328], p=0·41; figure 4). In regression analyses, methotrexate was not associated with lower cellular immunogenicity compared with healthy controls (β=–0·30 [95% CI −1·07 to 0·46], p=0·42) or compared with targeted biological therapy (β=0·07 [–0·57 to 0·70], p=0·84). There was no association between T cell response and the time interval from the first and second dose of vaccine (appendix p 10) or clear difference in clinical or demographic characteristics between individuals who did and did not mount a T cell response (appendix p 4)

## Discussion

In this longitudinal cohort of individuals with psoriasis, monotherapy with methotrexate or targeted biological drugs did not significantly impair serological or functional humoral responses at 14 days following the second dose of BNT162b2, including against the delta variant. The second vaccine dose resulted in seroconversion in all participants. Whereas all controls had detectable total T-cell responses following the second vaccine dose, 18 (29%) of 63 individuals taking therapeutic immunosuppression had no evidence of T-cell responses.

Our findings indicate that a second dose of BNT162b2 vaccine, delivered according to an extended interval regimen, is successful in inducing antibody responses in individuals receiving methotrexate and targeted biologics. However, compared with healthy controls, a lower proportion of patients taking immunosuppressants had detectable T-cell responses or evidence of a boost in T-cell immunity following the second dose.

Our interim analysis findings showed that a proportion of both the control and immunosuppressed cohort had undetectable T-cell responses to the first vaccine dose.^13^ Following a second dose, all individuals in the control group demonstrated T-cell responses, whereas a proportion of immunosuppressed patients still had undetectable responses. Our results, albeit in a cohort of limited size, suggest that cellular immunity might diminish over time in some immunosuppressed individuals despite a second vaccine dose. An absence of boosting in cellular immunity in the context of immunosuppression supports findings from the OCTAVE study, in which patients with inflammatory arthritis and inflammatory bowel disease (receiving varying immunosuppressant therapies) had similar interferon-γ responses after both the first and second vaccine doses.^14^ In another recent study of a heterogenous group of patients with immune-mediated inflammatory diseases, patients receiving methotrexate did not have an increase in CD8^+^ T-cell activation after two vaccine doses.^19^

The importance of generation and boosting of T-cell responses for vaccine effectiveness is incompletely understood. T-cell immunity can influence long-term humoral immune dynamics; emerging research indicates that robust induction of antigen-specific T cells by mRNA vaccines enables durable neutralising antibody responses.^20^ The generation of long-term antibody-mediated immunity depends on the germinal centre reaction,^21^ which requires cooperation between antigen-specific T cells and B cells. Tfh cells sustain germinal centre responses, and their importance in coordinating humoral immunity after vaccination was underscored in research showing that detection of Tfh responses before a second vaccine dose correlated with neutralising antibodies after the second dose.^20^ Spike-specific antibodies following two vaccine doses have been shown to wane over time in immunocompetent individuals,^22^ but the kinetics of antibody responses in immunosuppressed patients is unknown. Our data indicate that a higher proportion of patients receiving immunosuppression do not have detectable Tfh cell responses following the second vaccine dose compared with controls (despite demonstrating antibody responses at 14 days after second dose; appendix pp 3, 9). Hence, assessing the longevity of vaccine-elicited antibody responses in these immunosuppressed patients and their ability to generate robust antibody responses on antigen re-exposure is important and might help to inform public health policy on the timing of subsequent vaccine doses and ongoing need for risk mitigating measures to reduce exposure to SARS-CoV-2.

Our humoral data at 14 days after a second vaccination indicate 100% seroconversion in patients receiving immunosuppression and similar functional humoral responses to healthy controls. Although our interim findings suggested lower serological responses to the first vaccine dose in patients taking immunosuppression compared with controls, with impaired functional humoral responses in those on methotrexate,^13^ the current data indicate successful boosting of antibody responses in all patients. Our data align with emerging findings from the CLARITY-IBD study,^15^ showing that a second vaccine dose led to seroconversion in most patients treated with the TNF inhibitor infliximab. Although neutralising responses or drug-specific effects were not assessed in OCTAVE, robust seroconversion (98%) was also detected following the second vaccine dose in patients with inflammatory arthritis.^14^ In that study,^14^ lower median anti-spike IgG titres were identified in patients with immune-mediated inflammatory diseases compared with controls. It is currently unclear whether this could translate to lower levels of protection.^23^ Although not statistically significant, the data from our limited sample size also suggest numerically lower levels of anti-spike IgG in immunosuppressed patients, compared with controls. Similar trends were identified in other cohorts of patients with immune-mediated inflammatory diseases,^9^, ^19^, ^24^ and these findings have raised questions about whether dose modification of methotrexate or temporary discontinuation could enhance immune responses to COVID-19 vaccines. As this strategy was shown to be successful in promoting immunogenicity to the influenza vaccine,^25^ a 2 week interruption in methotrexate after the COVID-19 booster is currently under investigation in a multicentre, randomised controlled trial.^26^ Assessments of functional humoral immunity in this study (which were reassuringly similar among all groups in our dataset), T-cell immunity, and clinical effectiveness will be important.

As the pandemic progresses, assessing immune cross-reactivity with novel variants of concern following vaccination is vital. There is a relative paucity of data for vaccine immunogenicity against the highly transmissible delta variant,^27^, ^28^ which is now the dominant variant worldwide. Population-level data indicate that the risk of COVID-19-related hospital admission associated with the delta variant is approximately double that of the alpha variant, with risk particularly increased in those with multimorbidities.^16^ Our data suggesting that methotrexate or targeted biologics do not impair neutralising antibody capacity against the delta variant are reassuring; however, there remains an urgent need to correlate immune findings with clinical effectiveness data.

Our study cohort is relatively homogeneous, and each study group (methotrexate, targeted biologics, healthy controls) was well matched with respect to clinical and demographic characteristics. No participants had a previous history of COVID-19 infection, and all had baseline or prevaccination data available. As a result of previously described age-related heterogeneity in responses to COVID-19 vaccines,^29^, ^30^ we enrolled a relatively young cohort (average age 44 years). All patients had well controlled psoriasis, enabling an investigation of drug-specific, rather than disease-specific, effects of vaccination. The immunogenicity assays used are highly reproducible. However, the number of participants and duration of follow-up are limited, hence these data should be interpreted with caution. It is possible that differences would become statistically significant with a larger sample size. It is also unknown how thresholds of antibody and cellular responses, which are based on levels in unvaccinated individuals and hence set at a conservative level, relate to clinical outcome. Patients with psoriasis who were not receiving immunosuppressants were not included, and most participants were of White ethnicity. All participants received BNT162b2 and no other vaccine types were included. It is also important to acknowledge that we have not corrected for multiple hypothesis testing as this study was exploratory. Larger and longer-term multicentre studies with more diverse study populations are required to validate and extend our findings, particularly in relation to the impact of an extended interval vaccination on the evolution of vaccine immune responses in immunosuppressed cohorts over time. Although our healthy control cohort received their second vaccine dose on average 1 week earlier than the immunosuppressed patients, our analysis indicates that this difference in timing is unlikely to affect immunogenicity. Studies involving cohorts of patients with different immune-mediated inflammatory diseases are required to understand the generalisability of our treatment-specific findings across diseases.

Our findings contribute to an important evidence base for informing future public health policy on prioritising populations for additional vaccine doses. Pending clinical effectiveness data, the current study supports the prioritisation of patients with immune-mediated inflammatory diseases receiving therapeutic immunosuppression to receive a further vaccine dose, since a proportion of our immunosuppressed cohort had undetectable cellular responses following a second dose (in contrast to healthy controls). Although we detected robust serological and neutralising antibody responses (including against the delta variant) in individuals receiving methotrexate and targeted biological monotherapy 14 days after the second vaccine dose, the immune correlates of vaccine effectiveness remain to be determined. The association between virus neutralisation and protection warrants investigation, as does the identification of clinically relevant seroprotection titres and the contribution of T-cell responses to protection. Long-term durability of antibody responses in the context of absent cellular responses is uncertain and merits further study.

## Data sharing

We are happy to share data on request to the corresponding author.
