# Supplementary material for: Humoral and cellular immunogenicity to a second dose of COVID-19 vaccine BNT162b2 in people receiving methotrexate or targeted immunosuppression: a longitudinal cohort study
Source: Lancet Rheumatol. 2021 Nov 9;4(1):e42–52. doi: 10.1016/S2665-9913(21)00333-7 (PMC8577228; doi:10.1016/S2665-9913(21)00333-7)
Supplement: Supplementary appendix [file mmc1.pdf]

THE LANCET
Rheumatology

Supplementary appendix

This appendix formed part of the original submission and has been peer reviewed. We post it as supplied by the authors.

Supplement to: Mahil S, Bechman K, Raharja A, et al. Humoral and cellular immunogenicity to a second dose of COVID-19 vaccine BNT162b2 in people receiving methotrexate or targeted immunosuppression: a longitudinal cohort study. *Lancet Rheumatol* 2021; published online Nov 9. https://doi.org/10.1016/S2665-9913(21)00333-7.

Supplementary appendix

Appendix Table 1. Baseline characteristics of study participants with data following both the first and second dose of COVID-19 vaccine BNT162b2.

	HV		MTX		TNFi		IL17i		IL23i		Total	
Number in each group	F (n=7)	M (n=8)	F (n=6)	M (n=8)	F (n= 11)	M (n=8)	F (n=7)	M (n=7)	F (n=8)	M (n=12)	F (n=39)	M (n=43)
Age (year)†	27.0 (24.0-48.0)	38.9 (33.0-46.5)	52.0 (46.0-56.0)	47.5 (41.0-57.5)	31.0 (22.0-52.0)	45.5 (30.0-55.5)	39.0 (37.0-48.0)	45.0 (40.0-49.0)	46.0 (34.5-50.5)	54.5 (35.5-60.0)	40.0 (28.0-51.0)	46.0 (37.0-57.0)
BMI (mg/m^2)†	25.30 (21.8-31.6)	24.0 (21.7-27.0)	31.5 (28.0-41.9)	27.1 (25.9-28.5)	29.3 (24.5-31.4)	31.6 (29.6-34.9)	26.5 (23.7-28.3)	29.3 (25.2-34.3)	28.0 (23.7-30.5)	29.0 (27.4-35.3)	28.3 (23.7-31.4)	28.7 (26.5-31.9)
Ethnicity, n (%)												
White	6 (85.7%)	7 (87.5%)	6 (100.0%)	5 (62.5%)	11 (100.0%)	7 (87.5%)	6 (85.7%)	6 (85.7%)	7 (87.5%)	10 (83.3%)	36 (92.3%)	35 (81.4%)
Black	0	0	0	1 (12.5%)	0	0	0	0	0	0	0	1 (2.3%)
South Asian	1 (14.3%)	1 (12.5%)	0	2 (25.0%)	0	1 (12.5%)	1 (14.3%)	1 (14.3%)	1 (12.5%)	1 (8.3%)	3 (7.7%)	6 (14.0%)
Mixed	0	0	0	0	0	0	0	0	0	1 (8.3%)	0	1 (2.3%)
Disease severity measure (psoriasis area severity index)†	-	-	2.1 (0.8-3.9)	2.3 (1.0-3.8)	1.0 (0.0-3.8)	1.8 (1.0-3.0)	0.0 (0.0-0.4)	1.2 (0.4-1.8)	0.6 (0.0-2.8)	0.6 (0.0-3.0)	0.7 (0.0-2.8)	1.3 (0.4-3.0)
Concomitant psoriatic arthritis, n (%)	-	-	2 (33.3%)	0	0	3 (37.5%)	4 (57.1%)	3 (42.9%)	3 (37.5%)	3 (25.0%)	9 (23.1%)	9 (20.9%)
Proximity MTX/biologic to v1 (days)	-	-	3.5 (2.0-5.0)	3.5 (1.0-4.5)	8.0 (1.0-10.0)	6.0 (5.5-10.0)	18.0 (11.0-21.0)	19.0 (10.0-21.0)	44.0 (28.0-57.5)	38.5 (19.0-45.5)	10.0 (5.0-21.0)	10.0 (4.0-26.0)
Proximity MTX/biologic to v2 (days)	-	-	2.0 (2.0-3.0)	3.0 (1.0-5.5)	7.0 (3.0-9.0)	4.0 (0.0-6.0)	20.0 (8.0-22.0)	17.0 (9.0-23.0)	47.0 (19.0-70.5)	34.5 (26.0-43.5)	8.0 (3.0-22.0)	6.0 (3.0-28.0)
Time between vaccine doses (weeks)	11.0 (10.3-11.1)	11.0 (10.4-11.5)	10.0 (9.9-10.7)	9.5 (9.3-9.8)	9.0 (8.9-9.1)	10.4 (10.1-10.9)	9.1 (8.4-11.0)	9.9 (9.6-10.0)	9.9 (8.5-10.3)	10.1 (9.8-10.9)	9.9 (9.0-10.7)	10.0 (9.7-11.0)

All values are given as numbers (%) unless otherwise specified. † Median (IQR). HV, healthy volunteers; MTX, methotrexate; TNFi, TNF inhibitors; IL17i, IL-17 inhibitors; IL23i, IL-23 inhibitors; v1, vaccine dose 1; v2, vaccine dose 2.

Appendix Table 2: Adverse events following the second dose of COVID-19 vaccine BNT162b2, disaggregated by sex.

		HV		MTX		TNFi		IL17i		IL23i		Total	
Number in each group		F (n=7)	M (n=8)	F (n=6)	M (n=8)	F (n= 11)	M (n=8)	F (n=7)	M (n=7)	F (n=8)	M (n=12)	F (n=39)	M (n=43)
Any Adverse Event	Mild	5 (71.4%)	7 (87.5%)	4 (66.7%)	3 (37.5%)	7 (63.6%)	5 (62.5%)	6 (85.7%)	6 (85.7%)	4 (50.0%)	7 (58.3%)	26 (66.7%)	28 (65.1%)
	Moderate	0 (0.0%)	0 (0.0%)	1 (16.7%)	0 (0.0%)	0 (0.0%)	0 (0.0%)	0 (0.0%)	0 (0.0%)	2 (25.0%)	0 (0.0%)	3 (7.7%)	0 (0.0%)
Any Systemic Event	Mild	3 (42.9%)	5 (62.5%)	0 (0.0%)	2 (25.0%)	4 (36.4%)	3 (37.5%)	4 (57.1%)	2 (28.6%)	1 (12.5%)	2 (16.7%)	12 (30.8%)	14 (32.6%)
	Moderate	0 (0.0%)	0 (0.0%)	1 (16.7%)	0 (0.0%)	0 (0.0%)	0 (0.0%)	0 (0.0%)	0 (0.0%)	2 (25.0%)	0 (0.0%)	3 (7.7%)	0 (0.0%)
Chills	Mild	1 (14.3%)	1 (12.5%)	0 (0.0%)	0 (0.0%)	0 (0.0%)	0 (0.0%)	0 (0.0%)	0 (0.0%)	0 (0.0%)	0 (0.0%)	1 (2.6%)	1 (2.3%)
Fatigue	Mild	1 (14.3%)	3 (37.5%)	0 (0.0%)	0 (0.0%)	1 (9.1%)	3 (37.5%)	2 (28.6%)	2 (28.6%)	1 (12.5%)	2 (16.7%)	5 (12.8%)	10 (23.3%)
	Moderate	0 (0.0%)	0 (0.0%)	1 (16.7%)	0 (0.0%)	0 (0.0%)	0 (0.0%)	0 (0.0%)	0 (0.0%)	1 (12.5%)	0 (0.0%)	2 (5.1%)	0 (0.0%)
Fever	Mild	1 (14.3%)	0 (0.0%)	0 (0.0%)	0 (0.0%)	0 (0.0%)	0 (0.0%)	0 (0.0%)	0 (0.0%)	0 (0.0%)	0 (0.0%)	1 (2.6%)	0 (0.0%)
Headache	Mild	0 (0.0%)	1 (12.5%)	0 (0.0%)	2 (25.0%)	2 (18.2%)	0 (0.0%)	1 (14.3%)	0 (0.0%)	0 (0.0%)	0 (0.0%)	3 (7.7%)	3 (7.0%)
	Moderate	0 (0.0%)	0 (0.0%)	0 (0.0%)	0 (0.0%)	0 (0.0%)	0 (0.0%)	0 (0.0%)	0 (0.0%)	1 (12.5%)	0 (0.0%)	1 (2.6%)	0 (0.0%)
Malaise	Mild	0 (0.0%)	0 (0.0%)	0 (0.0%)	0 (0.0%)	2 (18.2%)	0 (0.0%)	1 (14.3%)	0 (0.0%)	0 (0.0%)	1 (8.3%)	3 (7.7%)	1 (2.3%)
	Moderate	0 (0.0%)	0 (0.0%)	0 (0.0%)	0 (0.0%)	0 (0.0%)	0 (0.0%)	0 (0.0%)	0 (0.0%)	2 (25.0%)	0 (0.0%)	2 (5.1%)	0 (0.0%)
Nausea	Mild	1 (14.3%)	0 (0.0%)	0 (0.0%)	1 (12.5%)	0 (0.0%)	0 (0.0%)	1 (14.3%)	0 (0.0%)	0 (0.0%)	0 (0.0%)	2 (5.1%)	1 (2.3%)
Any Local Event	Mild	5 (71.4%)	5 (62.5%)	5 (83.3%)	2 (25.0%)	7 (63.6%)	3 (37.5%)	4 (57.1%)	5 (71.4%)	5 (62.5%)	5 (41.7%)	26 (66.7%)	20 (46.5%)
Induration	Mild	0 (0%)	0 (0%)	0 (0%)	0 (0%)	0 (0%)	0 (0%)	2 (28.6%)	0 (0%)	0 (0%)	0 (0%)	0 (0%)	0 (0%)
Itch	Mild	0 (0%)	0 (0%)	0 (0%)	0 (0%)	0 (0%)	0 (0%)	0 (0.0%)	0 (0%)	0 (0%)	0 (0%)	0 (0%)	0 (0%)
Pain	Mild	4 (57.1%)	5 (62.5%)	5 (83.3%)	2 (25.0%)	6 (54.5%)	3 (37.5%)	0 (0.0%)	4 (57.1%)	4 (50.0%)	5 (41.7%)	21 (53.8%)	19 (44.2%)
Redness	Mild	1 (14.3%)	0 (0%)	0 (0.0%)	0 (0%)	0 (0.0%)	0 (0%)	2 (28.6%)	0 (0%)	0 (0.0%)	0 (0%)	1 (2.6%)	0 (0%)
Swelling	Mild	1 (14.3%)	0 (0%)	1 (16.7%)	0 (0%)	0 (0.0%)	0 (0%)	2 (28.6%)	0 (0%)	1 (12.5%)	0 (0%)	3 (7.7%)	0 (0%)
Tender	Mild	1 (14.3%)	2 (25.0%)	2 (33.3%)	0 (0%)	3 (27.3%)	0 (0.0%)	0 (0.0%)	1 (14.3%)	0 (0.0%)	1 (8.3%)	8 (20.5%)	4 (9.3%)
Warmth	Mild	0 (0%)	0 (0%)	0 (0%)	0 (0%)	0 (0%)	0 (0%)	0 (0%)	0 (0%)	0 (0%)	0 (0%)	0 (0%)	0 (0%)
Worsening Psoriasis	Yes	-	-	1 (16.7%)	1 (12.5%)	4 (36.4%)	0 (0.0%)	1 (14.3%)	0 (0.0%)	1 (12.5%)	0 (0.0%)	7 (17.9%)	1 (2.3%)

Appendix Table 3. Individual cytokine response following the first and second dose of COVID-19 vaccine BNT162b2 in study participants.

	IL-2 response		IL-21 response		IFNγ response	
**1st Vaccine Dose**						
Healthy controls	10/15	67% (38% to 88%)	8/15	53% (27% to 79%)	9/15	60% (32% to 84%)
Patients on immunosuppressants	54/67	81% (69% to 89%)	57/67	85% (74% to 93%)	43/67	64% (52% to 76%)
Patients on methotrexate	11/14	79% (49% to 95%)	13/14	93% (66% to 100%)	9/14	64% (35% to 87%)
Patients on TNF inhibitors	14/19	74% (49% to 91%)	15/19	79% (54% to 94%)	15/19	79% (54% to 94%)
Patients on IL17 inhibitors	12/14	86% (57% to 98%)	12/14	86% (57% to 98%)	7/14	50% (23% to 77%)
Patients on IL23 inhibitors	17/20	85% (62% to 97%)	17/20	85% (62% to 97%)	12/20	60% (36% to 81%)
	Total 64/82		Total 65/82		Total 52/82	
**2nd Vaccine Dose**						
Healthy controls	13/14	93% (66% to 100%)	14/14	100% (77% to 100%)	11/14	79% (49% to 95%)
Patients on immunosuppressants	43/63	68% (55% to 79%)	47/63	75% (62% to 85%)	42/63	67% (54% to 78%)
Patients on methotrexate	7/13	54% (25% to 81%)	8/13	62% (32% to 86%)	7/13	54% (25% to 81%)
Patients on TNF inhibitors	13/19	68% (43% to 87%)	15/19	79% (54% to 94%)	14/19	74% (49% to 91%)
Patients on IL17 inhibitors	8/13	62% (32% to 86%)	10/13	77% (46% to 95%)	8/13	62% (32% to 86%)
Patients on IL23 inhibitors	15/18	83% (59% to 96%)	14/18	78% (52% to 94%)	13/18	72% (47% to 90%)
	Total 56/77		Total 61/77		Total 53/77	

The threshold values for IL-2, IL-21 and IFNγ response were established as 14, 10 and 17 cytokine secreting cells per million PBMCs, respectively, at which the response was classified as positive.

Appendix Table 4. Clinical and demographic characteristics by cellular response.

	Vaccine Dose 1		Vaccine Dose 2	
	T cell response	No T cell response	T cell response	No T cell response
Number in each group	N=68	N=14	N=59	N=18
Age (year)†	43.5 (32.0-51.0)	44.5 (33.0-58.0)	41.0 (29.0-50.0)	51.0 (40.0-58.0)
Gender (male)	35 (51.5%)	8 (57.1%)	31 (52.5%)	11 (61.1%)
BMI (mg/m^2)†	28.3 (25.4-31.3)	29.1 (24.0-35.3)		
Ethnicity, n (%)				
White	58 (85.3%)	13 (92.9%)	52 (88.1%)	14 (77.8%)
Black	1 (1.5%)	0 (0.0%)	0 (0.0%)	1 (5.6%)
South Asian	8 (11.8%)	1 (7.1%)	7 (11.9%)	2 (11.1%)
Mixed	1 (1.5%)	0 (0.0%)	0 (0.0%)	1 (5.6%)
Disease severity measure (psoriasis area severity index)†	1.2 (0.3-2.3)	0.8 (0.6-1.1)	0.9 (0.0-2.1)	1.8 (0.6-3.2)
Concomitant psoriatic arthritis, n (%)	14 (20.6%)	4 (28.6%)		
Treatment group				
HV	10 (14.7%)	5 (35.7%)	14 (23.7%)	0 (0.0%)
MTX	13 (19.1%)	1 (7.1%)	8 (13.6%)	5 (27.8%)
TNFi	15 (22.1%)	4 (28.6%)	15 (25.4%)	4 (22.2%)
IL17i	13 (19.1%)	1 (7.1%)	8 (13.6%)	5 (27.8%)
IL23i	17 (25.0%)	3 (21.4%)	14 (23.7%)	4 (22.2%)
Time between vaccine doses (weeks)	-	-	10.0 (9.1-11.0)	9.9 (9.7-10.1)

† Median (IQR). HV, healthy volunteers; MTX, methotrexate; TNFi, TNF inhibitors; IL17i, IL-17 inhibitors; IL23i, IL-23 inhibitors.

Appendix Figure 1. Local and systemic adverse events reported within 14 days after the second COVID-19 vaccine BNT162b2 dose.

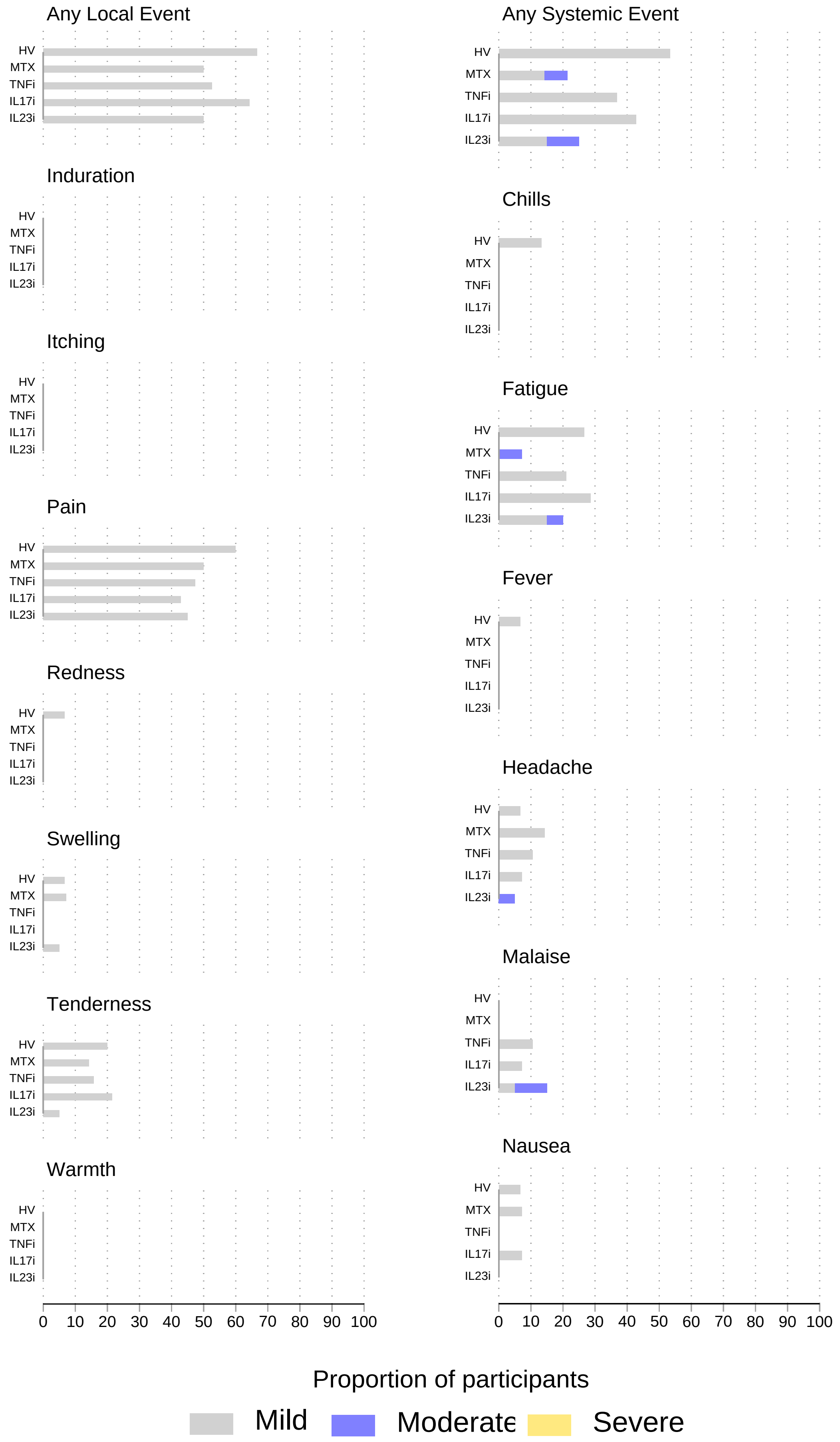

Proportion of participants in each study group reporting local and systemic adverse events following the second dose of COVID-19 vaccine BNT162b2. HV, healthy volunteers; MTX, methotrexate; TNFi, TNF inhibitors; IL17i, IL-17 inhibitors; IL23i, IL-23 inhibitors.

Symptoms were assessed according to the following scale: mild (no/minimal interference with activity and no/minimal medical intervention required), moderate (limitation in activity and medical intervention required), and severe (marked limitation in activity and medical intervention required).

Appendix Figure 2. Impact of time interval between vaccine doses upon neutralising antibody titres following the second COVID-19 vaccine BNT162b2 dose.

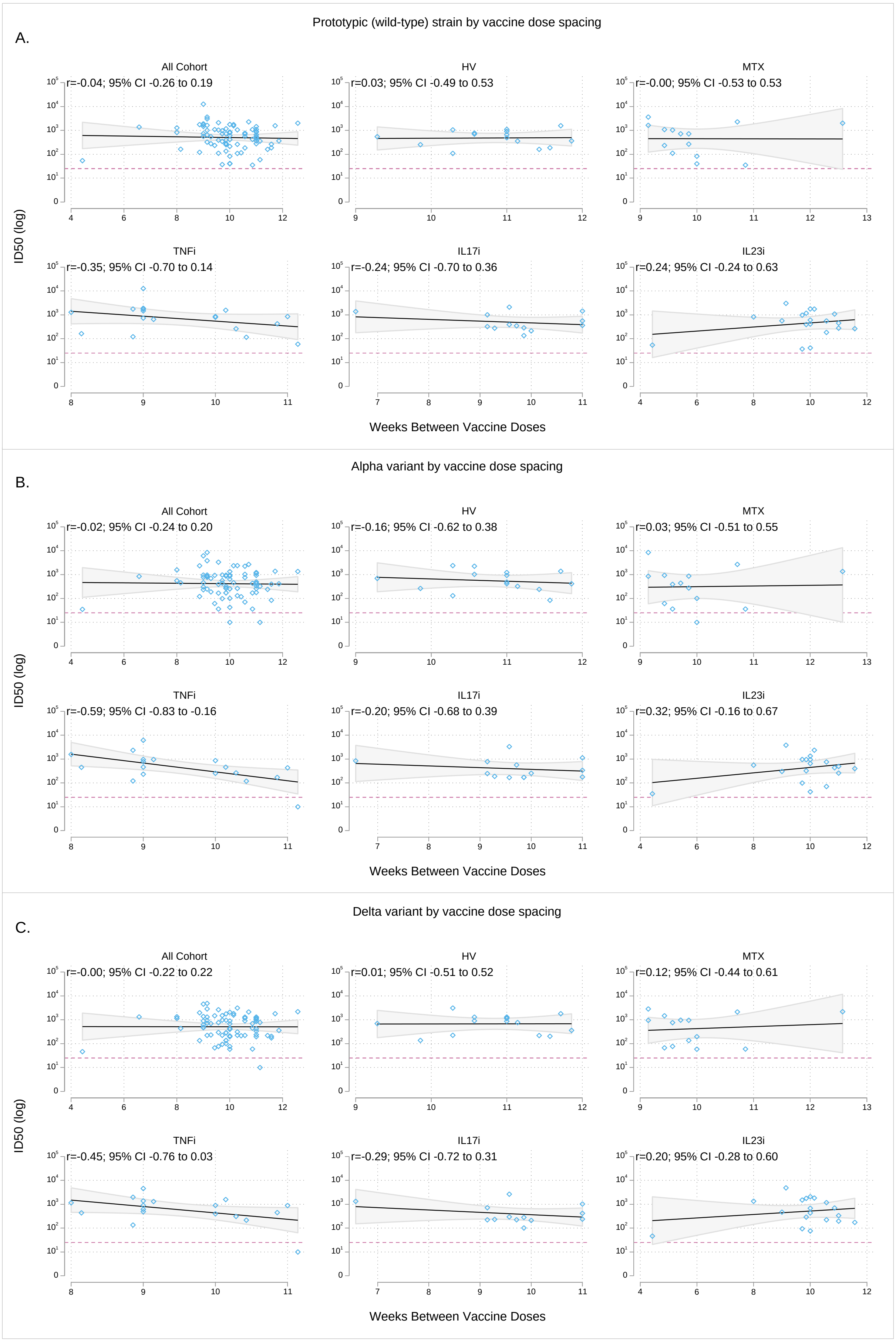

Appendix Figure 3. Total T cell response (IFNγ, IL-2 or IL-21) after the first and second dose of COVID-19 vaccine BNT162b2, by study participant.

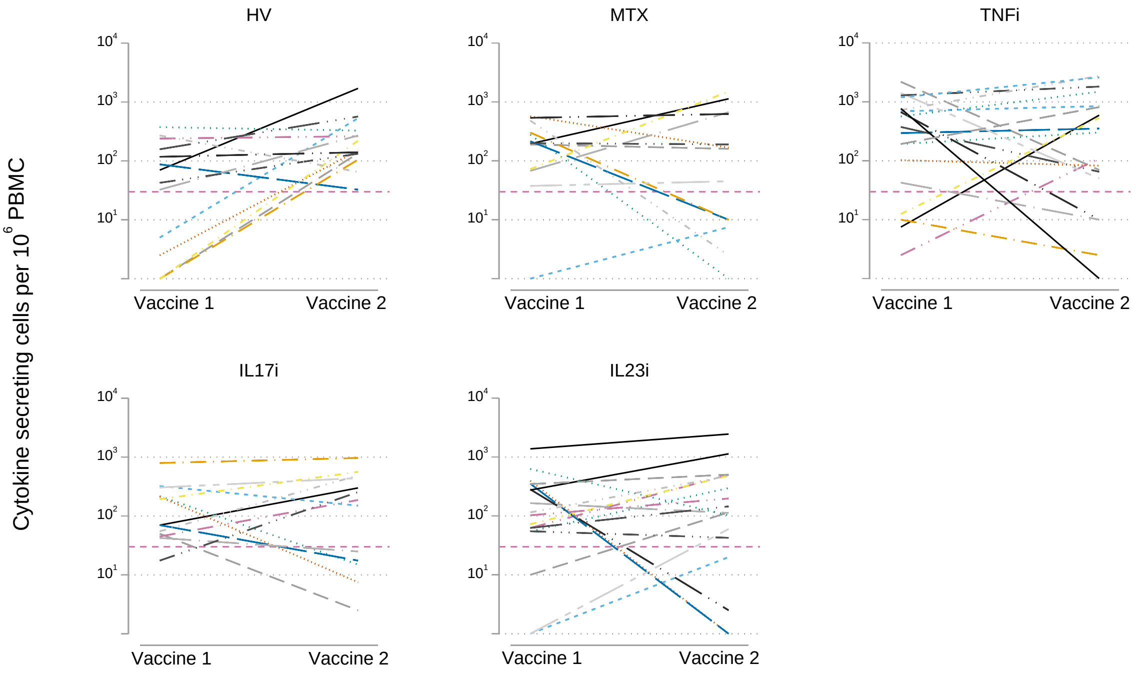

HV, healthy volunteers; MTX, methotrexate; TNFi, TNF inhibitors; IL17i, IL-17 inhibitors; IL23i, IL-23 inhibitors.

Appendix Figure 4. Individual cytokine response (IFNγ, IL-2, IL-21) after the first and second dose of COVID-19 vaccine BNT162b2.

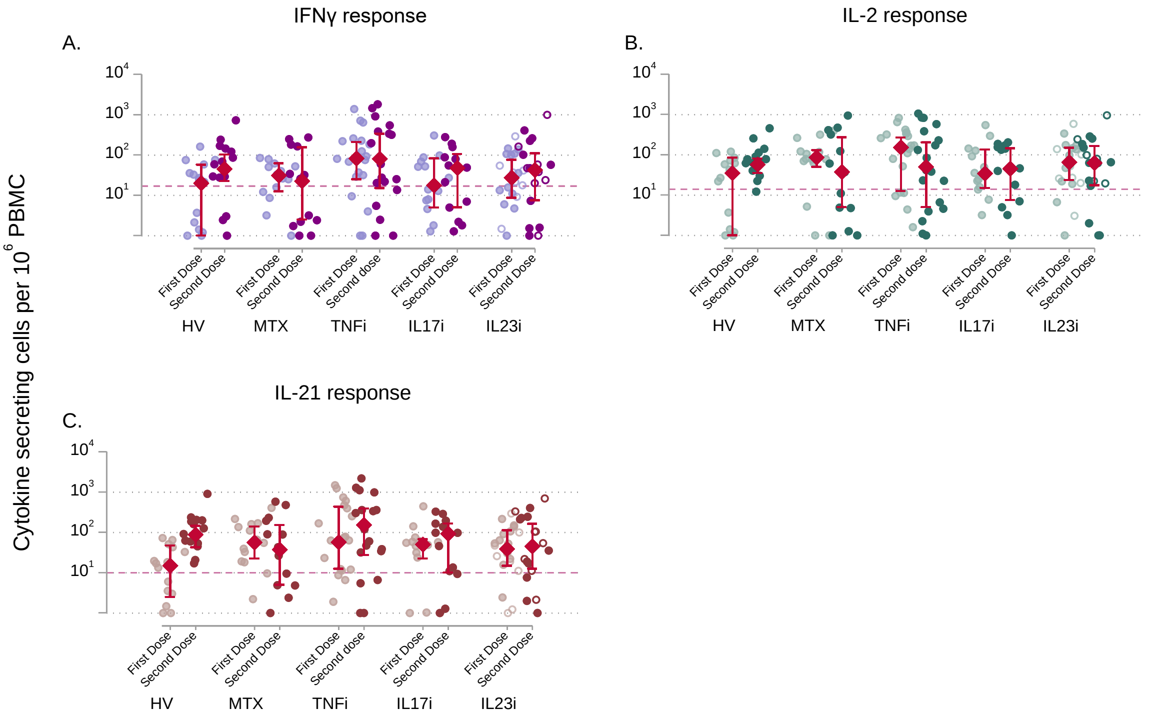

Individual cytokine response, as determined by **A)** IFNγ, **B)** IL-2 and **C)** IL-21 responses to stimulation with peptides from total Spike peptide pools, reported as number of cytokine secreting cells per 10^6 cells in PBMC samples at day 28 following the first dose and at day 14 following the second dose of COVID-19 vaccine BNT162b2. The circles represent individual values. The red diamonds and range lines indicate the median and interquartile range (IQR), respectively. Horizontal dashed lines indicate the cytokine response threshold. In the IL23i group, filled circles represent participants receiving IL-23p19 inhibitors and hollow circles represent participants receiving IL-23p40/IL-12 inhibition.

HV, healthy volunteers; MTX, methotrexate; TNFi, TNF inhibitors; IL17i, IL-17 inhibitors; IL23i, IL-23 inhibitors.

Appendix Figure 5. Impact of time interval between vaccine doses upon total T cell response following the second COVID-19 vaccine BNT162b2 dose.

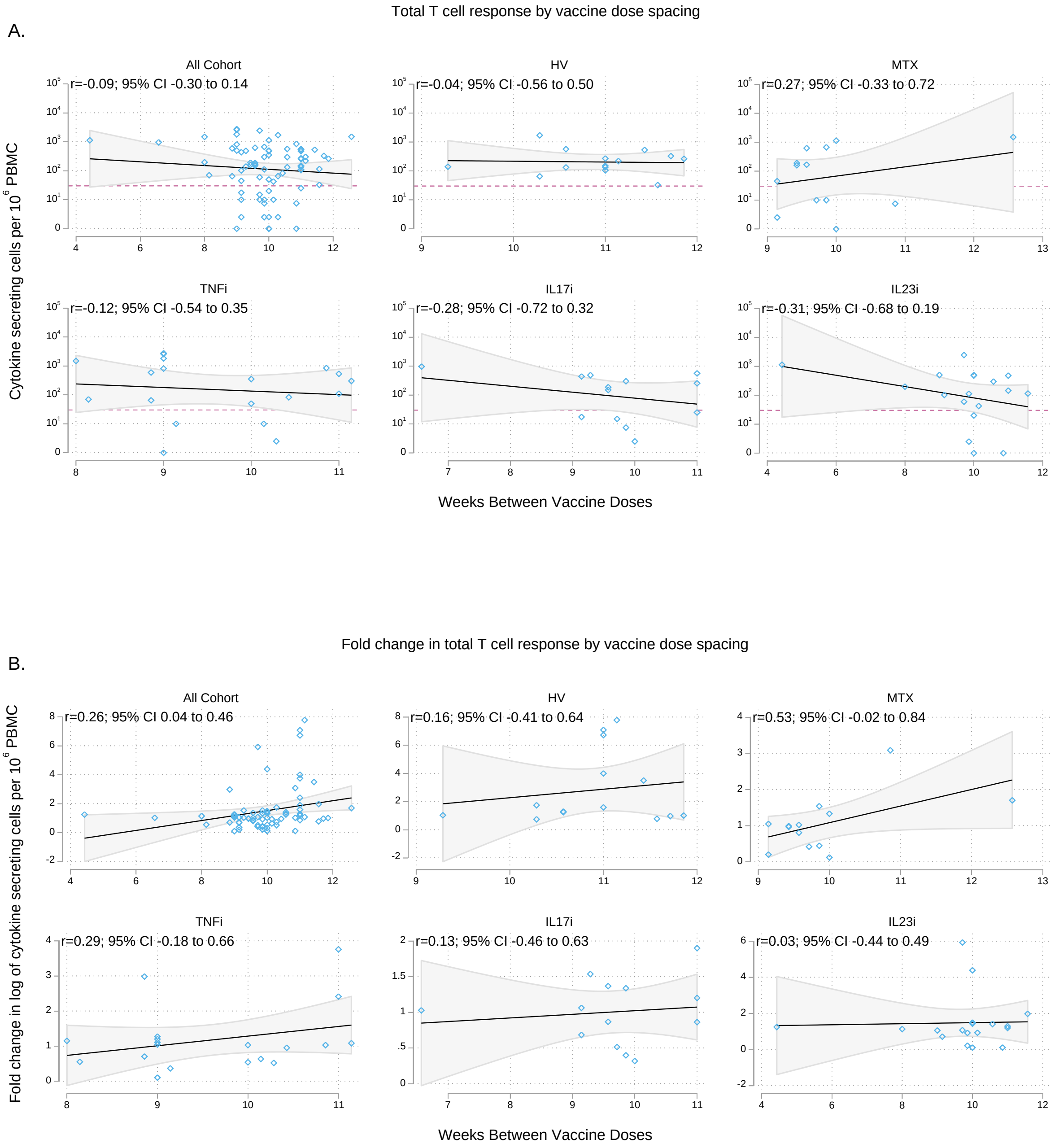

A. Spearman correlation between the time interval between the first and second doses of BNT162b2 and total T cell response post-second dose (cytokine secreting cells/106 PBMC).

B. Spearman correlation between the time interval between the first and second doses of BNT162b2 and the fold change in total T cell response post-first and second dose of BNT162b2.

HV, healthy volunteers; MTX, methotrexate; TNFi, TNF inhibitors; IL17i, IL-17 inhibitors; IL23i, IL-23 inhibitors.
